# Phenol and the Wasserman Reaction

**Published:** 1914-05

**Authors:** 


**Phenol and the Wasserman Reaction.** E. Signorelli, Zeit. fuer Immunitaetsforschung und Exp. Therapie, Vol. 19, section 2, finds that by diluting the serum ten times with physiologic salt solution containing ¼% phenol, the sensitiveness is increased. Whether this observation will lead to clearer cut diagnosis or whether it will give rise to non-specific reactions he has not determined.

